# Reported municipal costs from outdoor smoke-free by-laws-experience from Ontario, Canada

**DOI:** 10.1186/1617-9625-12-4

**Published:** 2014-02-28

**Authors:** Ryan David Kennedy, Dana Zummach, Stephanie Filsinger, Scott T Leatherdale

**Affiliations:** 1 Propel Centre for Population Health Impact, University of Waterloo, Waterloo, ON N2L 3G1, Canada; 2 Department of Health, Behavior & Society, Johns Hopkins Bloomberg School of Public Health, Institute for Global Tobacco Control, 2213 McElderry Street, 4th Floor, Baltimore, MD 21205, USA; 3 School of Public Health and Health Systems, University of Waterloo, 200 University Avenue West, Waterloo, ON N2L 3G1, Canada

**Keywords:** Municipal by-law, Outdoor smoking, Policy evaluation, Economic impact

## Abstract

**Background:**

In 2006, enclosed public and workplaces in Ontario were made smoke-free by the Smoke-free Ontario Act (SFOA). Numerous area municipalities across the province have since developed local by-laws that are more restrictive than the SFOA and ban smoking in outdoor environments including parks, beaches, and patios. The current study measured reported costs associated with the implementation and enforcement of smoke-free outdoor municipal by-laws including materials and staffing costs. The study also assessed the number of warnings or tickets issued to smokers. Ontario communities with a by-law in force for at least 2 years were included in the sample (n = 42). The study was completed by 88% of area municipalities (n = 37). Municipal staff and managers completed a survey by telephone between June-September 2012.

**Findings:**

No area municipality surveyed reported that they hired additional enforcement staff as a result of their community’s smoke-free by-law. Most municipalities (95%) posted signage to support awareness of their by-law; signs costs ranged from $40-$150/sign with most municipalities reporting signs were made in-house. Most communities reported actively enforcing the by-law; six communities reported they had issued tickets to people not in compliance with outdoor smoking restrictions.

**Conclusions:**

The implementation, promotion, and enforcement of outdoor smoke-free by-laws have required municipal staff time and in most cases have promotional costs, but these have come from existing budgets and using existing staff. Outdoor smoke-free by-laws have not created significant burdens on municipal enforcement staff or on municipal budgets.

## Findings

### Background

Reducing tobacco use remains a public health priority in Canada. It is estimated 17.3% of Canadians-approximately 4.9 million people-are current smokers [1]. The decline in smoking prevalence over the past decade appears to have slowed, suggesting more comprehensive policy responses are needed to support smokers to quit, deter new tobacco users, and protect people from exposure to secondhand smoke (SHS).

Enclosed workplaces and public places regulated by the province of Ontario were made smoke-free in 2006 by the Smoke-free Ontario Act (SFOA) [2]. Ontario has 444 area municipalities including towns, cities, and regions [3]. These area municipalities have the jurisdictional authority to enact tobacco control by-laws that are more stringent than the provincial law. Since 2006, dozens of communities across the province have developed and enacted by-laws that ban smoking in a variety of outdoor environments including parks, beaches, patios, doorways, and outdoor transit environments. The rationale for some of these policies is to further protect citizens from exposure to outdoor tobacco smoke (OTS) [4]. Studies have demonstrated that OTS concentrations can be similar to those measured indoors [5]. In addition to protection, some outdoor smoke-free policies were enacted to change social norms around smoking and support positive role modeling; these policies sought to make smoking less acceptable and subsequently prevent young people from initiating tobacco use [6].

An Ontario based Community-of-Practice working on advancing outdoor tobacco-free policies [7] identified that an important political barrier to the development and enactment of outdoor smoke-free by-laws is lack of evidence around by-law enforcement costs. This study sought to understand strategies used by local municipalities to promote their outdoor smoke-free policies, and measure financial impacts associated with the implementation and enforcement of by-laws in Ontario including materials and staffing costs. This study also wanted to understand to what extent these by-laws were being actively enforced, and if tickets or citations issued to people not in compliance with outdoor smoke-free by-laws were being challenged through municipal courts or other appeal mechanisms.

### Research hypothesis

The research team hypothesized that the majority of Ontario area municipalities with outdoor smoke-free by-laws invested financial and staff resources into the promotion of the new by-law. We further hypothesized that no new staff were hired to support the enforcement of outdoor smoke-free by-laws.

## Methods

### Sample

A non-government organization, the Non-Smokers’ Rights Association, maintains a database of Canadian policies related to tobacco control [8]. This database was reviewed and Ontario communities with by-laws more comprehensive than the SFOA were identified (n = 59). In order to accurately assess financial costs associated with by-law implementation and enforcement, communities included in the sample had by-laws that had been enacted a minimum of 2 years prior to the survey–meaning a by-law was enacted since June 1, 2010. This ensured that municipalities surveyed had experienced a minimum of one annual budget cycle since the by-law came into force. Based on this inclusion criterion, 42 communities were identified. These municipalities ranged in size from small towns (less than 1000 residents) to large cities (more than 500,000 residents). Most by-laws restricted smoking in doorways (as a minimum) and many further restricted smoking in parks, recreational fields, and on municipal beaches.

Municipal staff and managers responsible for the by-law responded to closed-ended survey questions administered over the telephone by a single researcher between June-September 2012. In most instances, multiple respondents were interviewed in each municipality including by-law officers and public health staff involved with health promotion.

The survey asked respondents to share number of resources and costs associated with outdoor smoke-free by-law promotion, and enforcement staffing. Respondents also reported the number of tickets or warnings issued to people not compliant with their by-law (since it was enacted), and the number of tickets that had been challenged in any dispute mechanism (such as a court) and any associated legal costs. See List of Survey questions (Table 1).

**Table 1:** Survey questions & surveyor script

**#**	**Question**	**Prompts**
*The first few questions ask about resources allocated towards bylaw promotion and awareness.*		
1.	Did the community use signs to communicate the new bylaw? How many? Do you know how much it cost to produce and post those signs?	
2.	Did staff do presentations? To whom?	
3.	Did the community use pamphlets, brochures and/or posters to communicate the new bylaw? How many? Do you know the cost to print and post these?	
4.	Did the community hold public meetings? Do you know how many? Was there a cost associated with these meetings?	
5.	Were any other resources used to promote the new bylaw?	What is the approximate number of each of the following resources that were allocated towards promotion and awareness of (Bylaw No.):
		◦ Signs?
		◦ Presentations?
		◦ Pamphlets/Brochures/Posters?
		◦ # of Public Town/City/Municipal Meetings held?
		◦ Other (Please Specify):
6.	What is the approximate cost of each of the following resources that were allocated towards promotion and awareness of (Bylaw No.):	◦ Signs?
		◦ Presentations?
		◦ Pamphlets/Brochures?
		◦ Public Town/City/Municipal Meetings held?
		◦ Other (Please Specify):
*The next few questions ask who is responsible for enforcing the outdoor smoke-free bylaw in your municipality, their enforcement approach, and the number of warnings and tickets that have been issued.*		
7.	In your municipality, who is designated to enforce the bylaw?	
	____ Bylaw Enforcement Officer for Municipality	
	____ Local police service	
	____ Tobacco Enforcement Officer from the Health Unit	
	____ Combination of both – Please explain:	
8.	The next question asks you to describe your municipality’s enforcement approach. Please indicate if your approach incudes any of the following:	
	_____ Routine inspections	
	_____ Responding to complaints	
	_____ Other – Please specify:	
9.	How many warnings have been issued since the implementation of (Bylaw No.)? Did your community have a set period of time where there was a policy of issuing warnings instead of tickets – or other grace period? [yes/no] – if yes, for how long?	[It’s possible many places had a phase-in period where they did warnings for some time? If this was the practice that should be identified]
10.	How many tickets have been issued since the implementation of (Bylaw No.)? How many of these tickets/fines have been challenged in municipal courts or other appeal processes?	
11.	In terms of resources allocated to your city/municipality’s enforcement staff: How many additional bylaw officers were hired as a result of the implementation of (Bylaw No.) in terms of Full-Time Equivalents (FTE)?	
12.	Did your municipality re-allocate resources initially to enforce this by-law during its roll-out?	
13.	How much additional cost was allocated towards enforcement staff (e.g. for hiring, salaries) as a result of the implementation of (Bylaw No.)?	
*The next few questions deal with public satisfaction and support for (Bylaw No.) including calls to the Tobacco Information Line or other methods of reporting complaints.*		
14.	For your health unit, how many calls have you received to the Tobacco Information Line to address public complaints associated with (Bylaw No.) or outdoor smoking in general?	Are you able to tell how me many of these complains are from multiple sources or the sample people calling multiple times?
		Have there been any calls to the Tobacco Information Line in support of (Bylaw No.)?
15.	Is there another method by which people can submit a complaint? If yes, what is this method?	How many complaints have been received by this method associated with (Bylaw No.) or outdoor smoking in general?

Human research ethics board approval was not needed for this project since respondents were reporting data associated with their jobs, and no personal opinions were solicited.

## Results

### Response rate

The survey was completed by 88% of area municipalities (n = 37). No area municipality refused to complete the survey; non-responses were due to difficulty coordinating interview times.

### Promotional activities

Area municipalities reported using a variety of strategies to promote their outdoor smoke-free by-law including signage, presentations at public meetings and the provision of promotional material including pamphlets, brochures or web content.

### Signage

**Figure 1:**
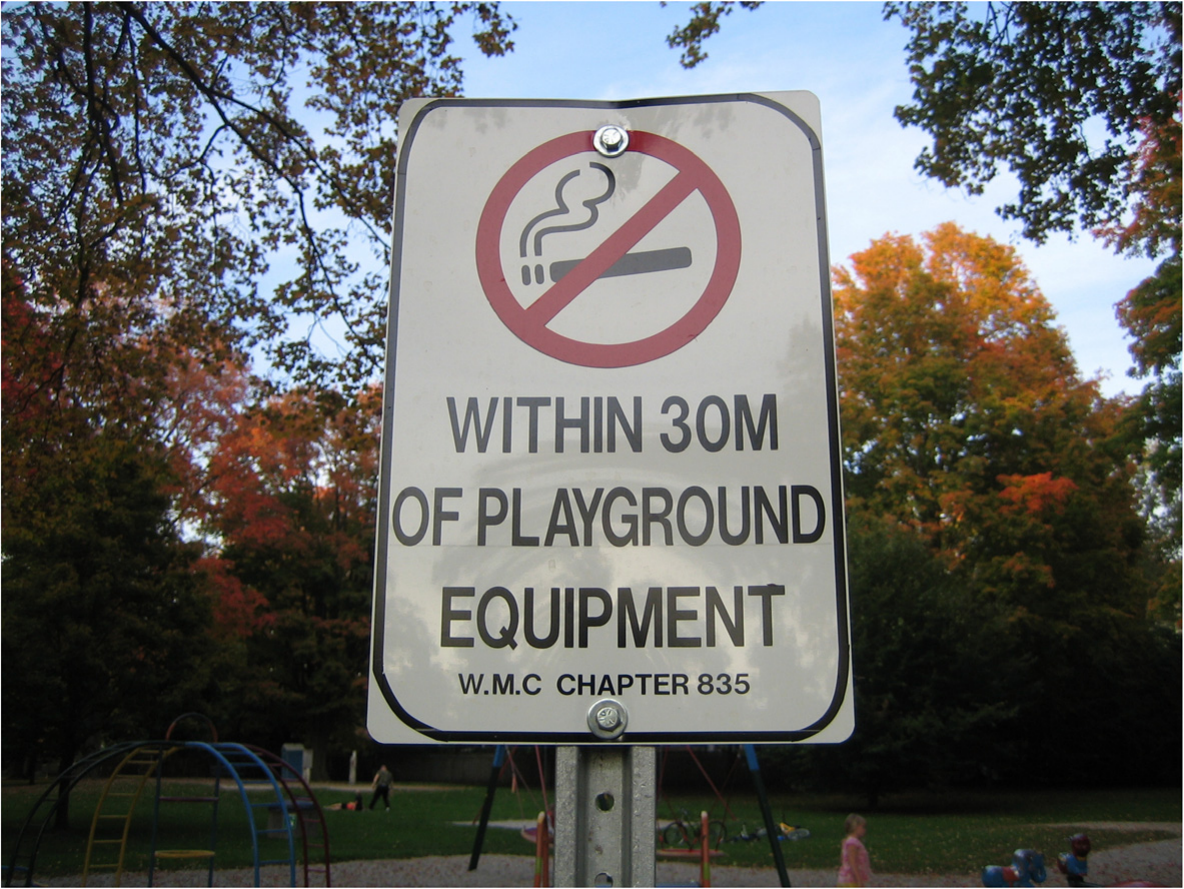
Sample sign used to promote municipal outdoor smoke-free by-law in Ontario community.

Almost all municipalities (n = 35; 95%) reported that they posted signage to support awareness of their outdoor smoke-free by-law. Most municipalities could not report on the total costs incurred to post signs as most towns reported producing signs in-house, and staff time to post signage was not tracked. Some respondents explained that signage was added to existing sign poles reducing cost/time. Six communities did purchase signs from external vendors; costs per sign ranged from $40-$150. See Figure 1 for an example of an outdoor sign produced to educate park users of the local by-law and the smoking restrictions.

### Presentations and public meetings

Respondents reported that most municipalities (n = 23; 62%) had staff who made presentations in the community about the by-law. The most commonly mentioned audiences were sports groups that used municipal recreation fields. The majority of municipalities (n = 30; 81%) reported holding at least one public meeting to introduce the new by-law. Respondents reported that presentations and public meetings did not require additional financial resources since there were no booking fees or rentals required; however, staff time was needed.

### Promotional material

Almost half of respondents (n = 15; 40%) reported that their municipality developed and printed promotional materials including pamphlets, brochures and/or posters to communicate the new by-law. Promotional materials were often produced and distributed in partnership with the local health unit (n = 10; 27%). Several municipalities included information about the by-law in recreation/tourism brochures (n = 5; 14%). Quantity and cost of producing the promotional materials were often unknown as they were produced in house or not recorded.

Other promotional efforts included: community newspaper ads, notices on municipal and health unit websites, social media campaigns, and bus ads. These promotions had costs but respondents reported these were covered by existing promotional budgets, using agreements with municipal run transit authorities, and/or standing media buys from the municipality with local newspapers.

### Enforcement

No area municipality reported that they hired additional enforcement staff as a result of their community’s smoke-free by-law. No respondent reported that their community allocated additional resources initially to enforce their by-law during its roll-out. Respondents reported that the majority of area municipalities (n = 28, 76%) enforce their by-law with municipal by-law enforcement officers; some area municipalities (n = 5, 14%) exclusively use tobacco enforcement officers who are health unit staff funded by the province. Other municipalities use a combination of enforcement staff, including municipal by-law officers, provincial tobacco enforcement officers, local police, and municipal parks and recreation staff.

### Ticketing

All respondents indicated that their area municipality responds to complaints of people smoking in environments covered by their outdoor smoke-free by-law. More than half of the respondents (n = 22, 59%) reported that their municipality conducts routine inspections or actively enforces the by-law some of the time. Since smoking is generally a visible activity, many respondents explained that by-law officers can look in at parks while they are driving or biking by areas. Respondents explained that by-law officers enforce all by-laws for their community and will “monitor locations when passing by”. Some respondents explained that routine inspections were conducted if frequent complaints were received from a specific location. Some common environments identified included sports complexes, arenas, and recreational fields. Some respondents shared how difficult it is to ‘catch’ a person smoking since a cigarette generally takes less than 10 minutes to smoke, and many of the communities surveyed have response times longer than that. More than half of the municipalities (n = 21, 57%) reported that they have issued warnings to people not compliant with their outdoor smoke-free by-law. Respondents from six communities (n = 6, 16%) reported that their area municipality has issued tickets to individuals smoking in restricted outdoor environments. Most of these communities (n = 4) have only issued 1 or 2 tickets. No municipality reported that they experienced contested tickets requiring court time.

## Conclusions and discussion

The findings from this study indicate that, across Ontario, outdoor smoke-free outdoor by-laws have been enacted and supported using pre-existing municipal resources including staff and operational budgets. No community reported that their outdoor smoke-free by-law resulted in the hiring of additional staff. Posting signage was a common promotional activity. Enforcement strategies differed by community but most municipalities reported that they actively enforce their outdoor smoke-free by-law at least some of the time. More than half of the respondents reported that warnings have been issued; however, only 6 respondents reported that their area municipality has issued tickets to people not in compliance with their by-law.

This study’s findings help answer questions about costs and activities associated with the implementation of outdoor smoke-free by-laws in Ontario. Elected municipal councils are often called on to balance a community’s desire to regulate behavior, with the need to be financially responsible with public budgets. The experience of the communities included in this study show that municipalities, and specifically enforcement officers were not overwhelmed with complaints from members of the community about people smoking in regulated outdoor spaces. This means that these by-laws did not result in the reallocation of significant human or fiscal resources away from other community by-laws and therefore represent a minimal cost to the municipality. It is not possible to conclude from this study the extent to which these by-laws are creating smoke-free spaces. It is clear that active enforcement of these by-laws could be greater; however, the initial findings with respect to warnings and tickets are promising.

This study has numerous limitations. Given that there was a wide range of regulation between by-laws–meaning some regulated doorways, while others regulated multiple environments, it is difficult to directly compare promotional activities or enforcement efforts. The study also relied on respondents to recall historic decisions and budget details that in some cases happened many years ago.

As more comprehensive by-laws are enacted in Ontario it will be important to further evaluate impacts on municipal operations including trends around warnings and tickets issued, and on compliance and overall behavior change.

## Abbreviations

NSRA: Non-Smokers’ Rights Association; OTS: Outdoor tobacco smoke; SFOA: Smoke-free Ontario Act; SHS: Secondhand smoke.

## Competing interests

The authors have no financial or other competing interests to declare.

Ryan David Kennedy was a member of the Community of Practice (CoP) that identified the need for this research.

Aspects of this paper were presented in a poster at the 19th Annual International Meeting of the Society for Research in Nicotine and Tobacco (SRNT) at the Westin Waterfront Hotel in Boston MA in 2013.

## Authors’ contributions

RDK and SL developed the research questions and methods, and survey tool. RDK was the lead author on the paper. DZ conducted the survey and summarized the findings. DZ also assisted with reviewing manuscript drafts. SF supported the project and assisted with manuscript preparation and submission. SL also provided comments and edits on the manuscript. All authors read and approved the final manuscript.
